# Where Do the Electrons Go? Studying Loss Processes in the Electrochemical Charging of Semiconductor Nanomaterials

**DOI:** 10.1021/acs.chemmater.4c02998

**Published:** 2025-01-13

**Authors:** Reinout F. Ubbink, Yan B. Vogel, Maarten Stam, Hua Chen, Arjan J. Houtepen

**Affiliations:** Optoelectronic Materials Section, Faculty of Applied Sciences, Delft University of Technology, Van der Maasweg 9, 2629 HZ Delft, The Netherlands

## Abstract

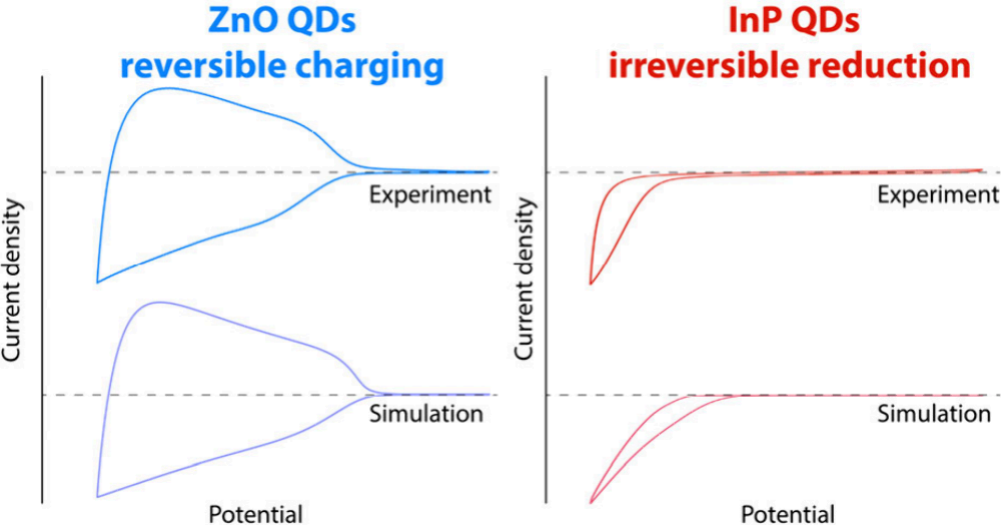

Electrochemical charging of films of semiconductor nanocrystals (NCs) allows precise control over their Fermi level and opens up new possibilities for use of semiconductor NCs in optoelectronic devices. Unfortunately, charges added to the semiconductor NCs are often lost due to electrochemical side reactions. In this work, we examine which loss processes can occur in electrochemically charged semiconductor NC films by comparing numerical drift-diffusion simulations with experimental data. Both reactions with impurities in the electrolyte solution, as well as reactions occurring on the surface of the nanomaterials themselves, are considered. We show that the Gerischer kinetic model can be used to accurately model the one-electron transfer between charges in the semiconductor NC and oxidant or reductant species in solution. Simulations employing the Gerischer model are in agreement with experimental results of charging of semiconductor NC films with ideal one-electron acceptors ferrocene and cobaltocene. We show that reactions of charges in the semiconductor NC film with redox species in solution are reversible when the reduction potential is in the conduction band of the semiconductor NC material but are irreversible when the reduction potential is in the band gap. Experimental charging of semiconductor NC films in the presence of oxygen is always irreversible in our system, even when the reduction potential of oxygen is in the conduction band of the semiconductor NC material. We show that the Gerischer model in combination with a coupled reversible-irreversible reaction mechanism can be used to model oxygen reduction. Finally, we model irreversible reduction reactions with the semiconductor NC material itself, such as reduction of ligands or surface ions. Simulations of semiconductor NC cyclic voltammograms in the presence of material reduction reactions strongly resemble experimental cyclic voltammograms of InP and CdSe NC films. This marks material reduction reactions at the semiconductor NC surface as a likely candidate for the irreversible behavior of these materials in electrochemical experiments. These results show that all reduction reactions with redox potentials in the band gap of semiconductor NCs must be suppressed in order to achieve stable charging of these materials.

## Introduction

The ability to controllably charge films of semiconductor nanocrystals like quantum dots (QDs), nanoplatelets or nanowires with electrons or holes opens up possibilities for use of these materials in light-emitting electrochemical cells, photodetectors and lasers.^1−5^ Through electrochemical methods, the Fermi energy and thus charge density in semiconductor nanocrystal (NC) films can be controlled with great precision.^6−9^ When charging a semiconductor NC film electrochemically, a potential is applied to the film while it is submerged in an electrolyte solution containing free ions.^10^ The application of the potential leads to a flow of electrons or holes into the semiconductor NC film, depending on the sign of the applied potential. The charge of the additionally injected electrons/holes is compensated by ions flowing into the pores in the film from the electrolyte solution, resulting in electrochemical charging of the semiconductor NC film. In the ideal case, electrochemical charging of semiconductor NCs is fully reversible, meaning every electron/hole that is injected into the film remains there and can subsequently be extracted by returning the potential back to the open circuit potential and letting the charges flow back into the electrode.

Charging of semiconductor NC films is rarely fully reversible, however, and large differences in reversibility of charging are observed between different materials. For nanocrystals of materials like indium phosphide, cadmium selenide^11^ or lead halide perovskites,^12^ charges injected in the NCs are often lost, after which they cannot be extracted again. We have shown previously for ZnO and PbS NCs that even if charges can be recovered in a fast cyclic voltammetry experiment, they are lost on longer time scales.^9,13,14^ Methods have been developed to improve the stability of the injected charges,^8,15^ but these are hampered by the fact that it is not well understood what processes are responsible for the loss of charge density in doped semiconductor NC film. While electrochemical measurements on semiconductor NCs are typically executed under stringent oxygen- and water-free conditions, contaminations of these molecules in the electrolyte cannot be fully excluded, and both can be reduced at high enough negative potentials, resulting in loss of electrons. On the other hand, we can also not exclude that other redox active impurities are present, for instance on the surface of the semiconductor NCs, such as ligands, adsorbed water or OH- ions or unknown reaction products from the semiconductor NC synthesis and processing. Indeed, Pu et al. have shown that metal-carboxylate ligands are likely reduced in QLEDs, resulting in a drop in efficiency.^16^

In order to improve the stability of electrochemically injected charges into semiconductor NC films, we aim to identify which processes are most likely to results in loss of charges, and to what extent they occur in films of different semiconductor NC materials. To tackle this problem systematically, in this work we start by examining one-electron transfer reactions between the semiconductor NCs and redox molecules with variable reduction potential. Although there is no theoretical difference between charging the conduction band (CB) with electrons or the valence band with holes, we will focus on electron injection into the conduction band and loss of electrons, since this process is much more readily achieved in most semiconductor NC materials and hence better understood. The question ‘where do the holes go?’ is even harder to answer. This will be the topic of future publications. Since ZnO QD films can show nearly reversible charging behavior, we use them as a model experimental system to show how the charging is affected by electrochemical reactions between electrons in the QDs and different redox-active species in solution. We model the same systems using drift-diffusion simulations. In previous work we showed that the electrochemical charging of semiconductor NC films can be accurately simulated by tracking the movement of all mobile species using drift-diffusion simulations,^17^ however only ideal charging of semiconductor NC films was considered. Here we extend the simulations by including electrochemical reactions using the Gerischer kinetic theory.^18^

First we show how the redox potential (*E*^*0*^) of the redox-active species determines the reversibility of one-electron transfer reactions in both simulation and experiment. Species with a reduction potential in the bandgap of the semiconductor NCs lead to irreversible loss of charges from the semiconductor NCs. While species with a reduction potential inside the conduction band also partake in electrochemical side-reactions, these are fully reversible and thus do not lead to irreversible loss of charges. This is shown experimentally by comparing cyclic voltammograms on ZnO QD films with either cobaltocenium (*E*^*0*^ inside the CB) or ferrocenium (*E*^*0*^ in the bandgap) added to the solution. Simulations show that when *E*^*0*^ is well below the CB, the reverse reaction (the oxidation of cobaltocene/ferrocene) is impeded, because it would only occur at much more positive potentials, resulting in irreversible loss of electrons.

Subsequently we address the more complicated case of reactions with molecular oxygen, which may give rise to subsequent chemical reactions between the formed superoxide molecules and ligands or protic impurities. Experiments show charging of ZnO QDs in oxygen-saturated solution are fully irreversible, even though the reduction potential of oxygen reduction is in the ZnO conduction band. Through simulations we show that this can be explained if the reduction of molecular oxygen to superoxide is followed by an irreversible chemical reaction in a reversible electrochemical-irreversible chemical (E_r_C_i_) mechanism.

Finally we model reactions were the semiconductor NC material itself is reduced. While this could include bulk decomposition of the materials, bulk decomposition is rarely observed in the potential ranges discussed here. More likely are reactions with ligands or surface ions on the surface of the NCs, as the most reactive species reside on the surface. It is found that the ability to reversibly charge a material depends on the energy of the CB edge compared to the lowest *E^0^* of the available reduction reactions that can occur in that material. Simulations of material reduction reactions closely resemble experimental data of InP and CdSe QDs, marking material reduction reactions as a likely candidate for electron loss in these materials.

Taken together, these results show that the stability of electrochemical charges in semiconductor NC materials is determined by electrochemical side reactions and their reduction potentials in relation to the energy of electrons in the CB. All of these reactions must be suppressed to achieve stable and reversible electrochemical charging of semiconductor NCs. Based on experiments and simulations, we provide strategies for improving the reversibility and stability of electrochemical charging of semiconductor NCs.

## Results and Discussion

### Reversible and Irreversible Charging of QD Films

**Figure 1:**
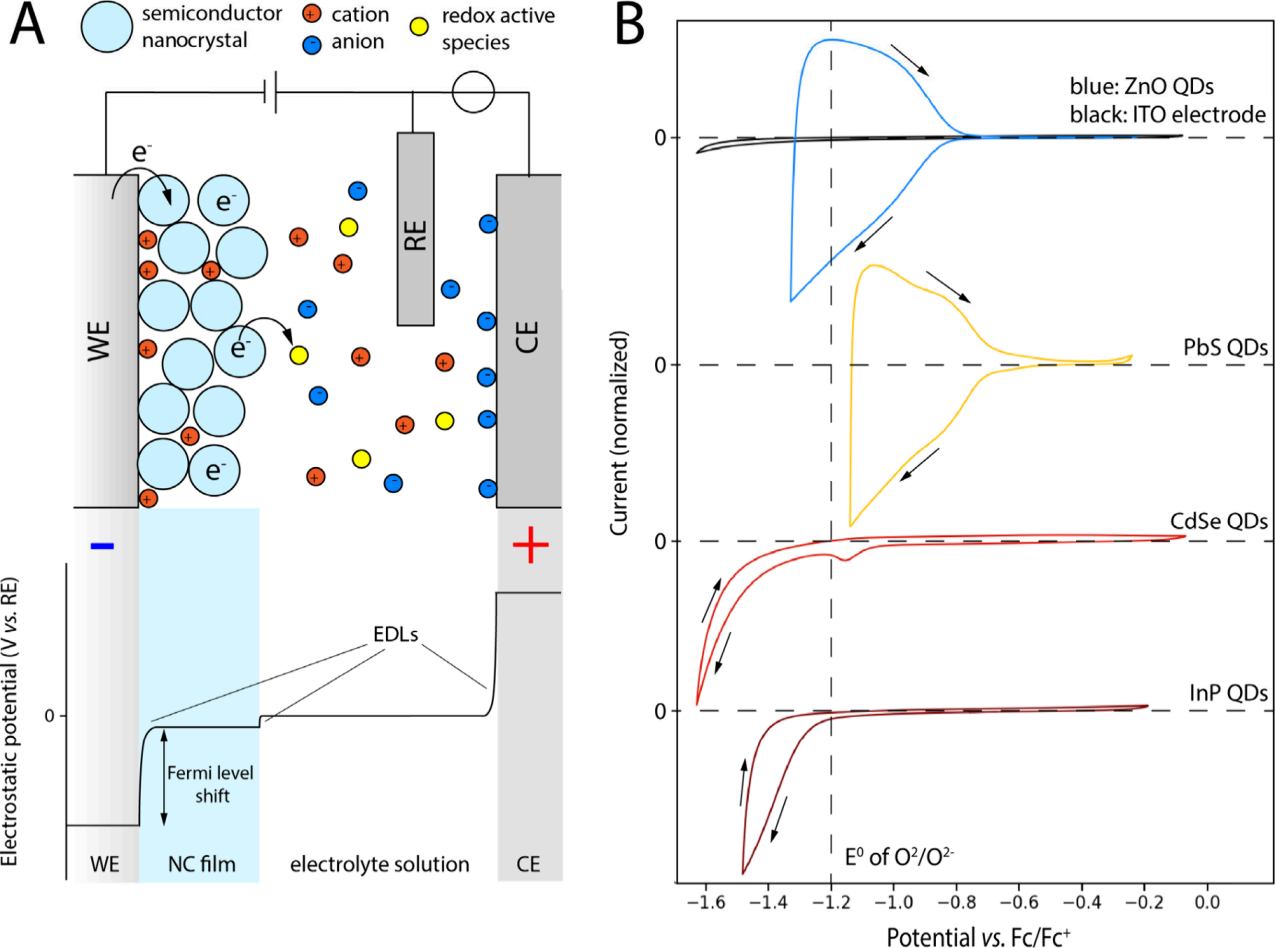
(A) Schematic of the setup used for electrochemical charging of semiconductor NCs. Semiconductor NCs are deposited on the working electrode (WE) and placed in an electrolyte solution containing ions in a three-electrode system with a reference electrode (RE) and counter electrode (CE). The electrostatic potential drops due to the formation of electric double layers (EDLs) at the interfaces between WE/film, film/solution, and solution/CE. (B) Cyclic voltammograms of QD films, normalized to the peak cathodic current. QD sizes, film thickness, and ligand types can be found in Table S1 in the Supporting Information and in the Methods section. All materials are measured with 0.1 M LiClO_4_ in acetonitrile as the electrolyte at a scan speed of 50 mV/s. Scan directions are indicated by arrows. Almost reversible charging is observed for ZnO (blue) and PbS (yellow) QDs (extraction ratios of 0.85 and 0.87, respectively), while CVs of CdSe (red) and InP (dark red) are almost fully irreversible (extraction ratios 0.1 and 0.08, respectively). Any observed positive current is ascribed to the formation and breakdown of the EDL on the electrode, which can also be observed for the bare indium tin oxide (ITO) WE (black).

The electrochemical setup of both experiments and simulations is shown schematically in Figure 1A. semiconductor NCs are deposited on a working electrode (WE), which is submerged in an electrolyte solution containing ions and optionally redox-active species which can react with charges in the semiconductor NC film. A three-electrode setup is used, where a voltage is applied to the WE with respect to a known reference electrode (RE), while the potential at the counter electrode (CE) is allowed to float to complete the circuit. When applying a potential to the semiconductor NC film in the presence of a sufficiently concentrated electrolyte solution, the ions will form an electric double layer (EDL) at the WE/NC film interface (Figure 1A), which will cause an increase of the electrochemical potential (μ̃) inside that semiconductor NC film by an amount approximately equal to the applied potential.^17^ Electrons will start to transfer into the semiconductor NC film if μ̃ is raised higher than the first available empty state in the CB of the semiconductor NCs. This results in an injection current into the semiconductor NCs. Counterions (cations in the case of electron injection) will migrate from the solution into the pores of the semiconductor NC film to compensate for the added charge, resulting in a net doping of the semiconductor NCs. In the ideal case, raising the applied potential further above the CB edge will cause any states available in the density of states (DOS) of the material up to μ̃ to fill up. Then when the applied potential is reduced again, μ̃ drops, electrons (or holes in case of positive potentials) will leave the QDs and ions will diffuse back into the solution. In the ideal case, electrons/holes would flow back into the electrode, resulting in an extraction current with opposite sign to the injection current.

This process of raising and lowering the potential can be done experimentally by recording a cyclic voltammogram (CV), where the potential is swept back and forth with a certain set scan rate. Figure 1B shows CVs performed on ZnO, PbS, CdSe and InP NC films. The CVs start at open circuit potential (OCP) and are scanned to the negative vertex first and then back to OCP (indicated with arrows) at a scan rate of 50 mV/s. In the experimental CV on ZnO QDs, the aforementioned injection and subsequent extraction of electrons can clearly be observed: as the applied potential becomes more negative, a negative current is observed, because electrons are injected into the material. The more negative the potential becomes, the higher μ̃ in the QDs is, and the more conduction states are filled with electrons. When scanning back, a positive extraction current is observed. By integrating the negative and positive parts of the scan, the extraction ratio can be determined for this material, defined as extracted charge/injected charge. For ZnO and PbS QD films (blue and yellow lines in Figure 1B), extraction ratios of >0.85 are observed, which indicates that the doping of the QDs is almost fully reversible. However, for other materials, extraction ratios are typically much lower. For films of CdSe and InP QDs (red and dark red lines in Figure 1B), very little or no extraction current is observed from these experiments (extraction ratios <0.1). Even though electrochemical doping takes place in these films, the process is irreversible. These results are typical. In the literature charge extraction from CdSe core-only QDs is rarely observed,^19^ and extraction ratios are low unless the temperature is reduced to −60 °C.^20^ For InP QDs, no charge extraction in cyclic voltammograms has been reported to the best of our knowledge.

Figure 1 shows only CVs to negative applied potentials. While electrochemical injection of holes into QDs should in theory be possible by applying positive potentials, this has been very challenging to achieve and has hardly been investigated except for a small number of studies on lead chalcogenide and CsPbBr_3_ perovskite nanocrystals.^21−23^ We will therefore focus on results of electron injection in this work and extend our discussion to hole injection later on.

### Loss of Electrons

**Figure 2:**
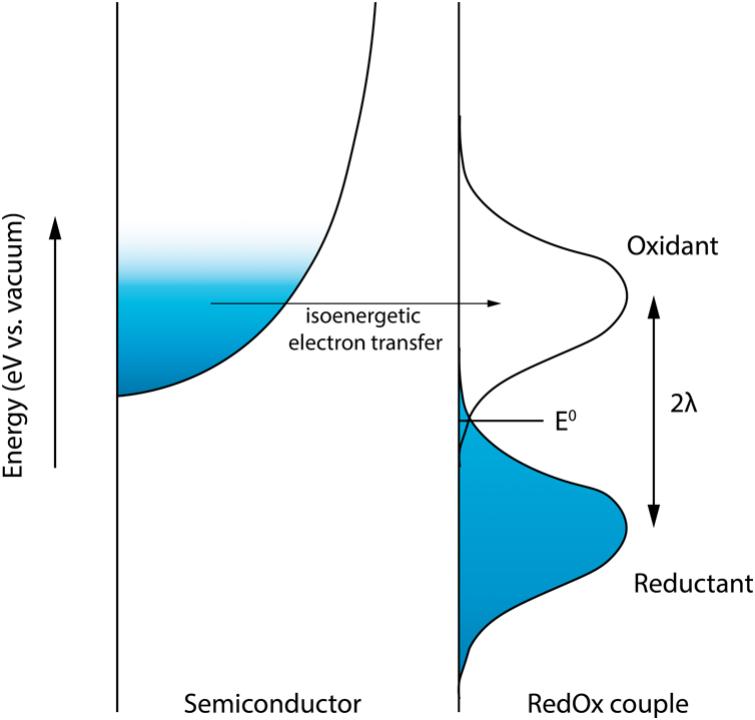
Example energy state distribution in the Gerischer kinetic model; filled states are blue and empty states are white. The semiconductor conduction band is modeled using a density of states function. The energy states of the redox couple are modeled using a redox potential *E*^*0*^ and a reorganization energy λ. The total electron transfer rate is calculated by integrating the rates of isoenergetic electron transfer over all energies.

When no extraction current is observed this implies that the injected electrons are no longer available for extraction, indicating “loss” of electrons from the semiconductor NCs. In CdSe and InP QDs the conduction band is higher in energy than in ZnO and PbS QDs. This means higher potentials need to be applied to achieve electron doping in these materials, and it becomes more likely that the reduction potential of reactions with impurities or on the surface of the semiconductor NC material itself is below the CB edge. To achieve reversible or even permanent^8,9^ doping in CdSe and InP QD films, it is important to understand what loss reactions can occur during the electrochemical doping of films. By simulating various electrochemical loss reactions and comparing to experimental data, we gain insight in what kinds of loss processes are occurring, and how to explain the discrepancies in doping reversibility of different QD materials. For this reason, we extended our earlier reported drift-diffusion simulations of electrochemical doping of QD films to include electrochemical side-reactions based on Gerischer kinetics (Figure 2, see Supporting Information for a full derivation and implementation).

In the Gerischer model,^18^ the rate of isoenergetic electron transfer between the semiconductor NCs and the redox-active species is calculated for each energy level separately. The total rate is then calculated by integrating over all energy levels, correcting for the occupancy of states in the semiconductor NC DOS using the Fermi–Dirac distribution. While for metallic working electrodes this is not always important, it is essential to take into account the DOS for semiconductors, as no reactions can take place from the energies in the band gap range. The energy states of the oxidant/reductant couple are modeled as Gaussian distributions using a redox potential *E*^*0*^ and a reorganization energy λ, as shown in Figure 2. The final parameter in the Gerischer model is the prefactor of the reaction rate *k*^*0*^, which includes the transfer attempt frequency and dimension correction factors (detailed explanation in the Supporting Information). By adjusting the value of these parameters, different types of redox species are modeled. The Gerischer model is identical to the Marcus model for transfer of an electron from a single energy state to an oxidant/reductant couple. However, since the Gerischer model takes into account electron transfer from all available energy states in the semiconductor NC DOS it is a better fit for semiconductor materials.

### Loss of Electrons to Reversible Oxidant in Solution

**Figure 3:**
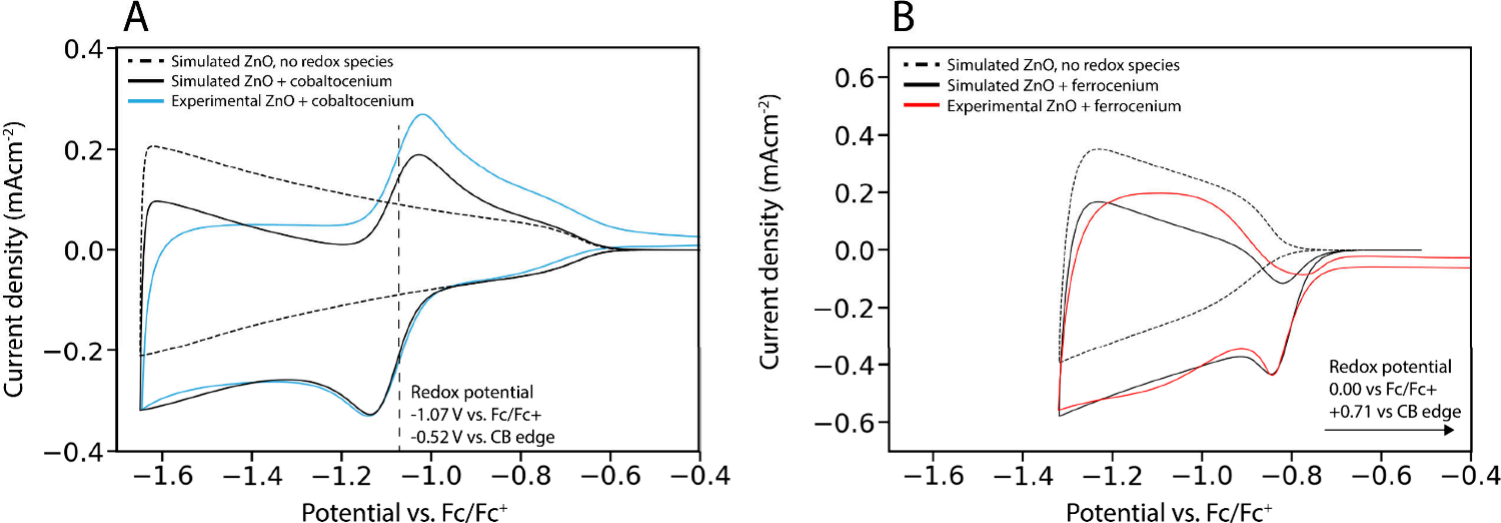
Experimental and simulated CVs of ZnO QD films in the presence of a redox couple at a scan rate of 50 mV/s. In the experiment, the electrolyte was acetonitrile containing 0.1 M LiClO_4_ and 1 mM of cobaltocenium/ferrocenium hexafluorophosphate. The QD diameter is 2.5 nm. (A) If the reduction potential is in the CB of the ZnO QDs, a reversible redox reaction is observed. The total current is the sum of the charging current in the ZnO QD film (dashed line) and the resulting redox current of the reduction reaction. (B) If the reduction potential is in the band gap of the ZnO QDs, an irreversible reduction current is observed as soon as the applied potential is raised beyond the conduction band edge.

To systematically investigate the effect of oxidants present in the electrolyte solution, we first investigate loss of electrons to an ideal dissolved oxidant. Cobaltocenium is known to undergo ideal one-electron reduction to cobaltocene, with an *E*^*0*^ at around −1.2 eV vs. Fc/Fc^+^ in acetonitrile, well inside the conduction band of the ZnO QDs (CB edge at −0.7 eV vs. Fc/Fc^+^). Figure 3A shows the experimental CV of a ZnO QD film submerged in an electrolyte solution containing 1 mM cobaltocenium (blue line). In addition to a charging current of electrons entering the ZnO CB starting around −0.7 V vs. Fc/Fc^+^, a clear reduction peak of cobaltocenium is observed at −1.1 V vs. Fc/Fc^+^. Oxidation of cobaltocene back to cobaltocenium is also observed on the backward scan as a positive peak, confirming that the reaction is reversible.

We performed simulations of the same system both with and without the inclusion of side-reactions with cobaltocenium, which are shown in Figure 3A (black lines). In the simulated scan without any oxidant present, perfect cyclical charging and discharging of ZnO is observed, with an extraction ratio of unity. The scan with the oxidant present in the simulation shows an additional reduction (negative current) and oxidation (positive current) peak around the reduction potential of the oxidant.

The resulting CV is the sum of the charging of the ZnO film and the expected ideal reduction/oxidation current of the redox couple. Although both processes are ideally reversible, the observed extraction ratio in both the experiment and simulation is less than unity (0.37 for simulation, 0.61 for experiment) because of a leak current: cobaltocene is formed at the working electrode, transported to the counter electrode through diffusion, and subsequently oxidized to form back cobaltocenium. This results in a net transport of electrons from the WE to the CE and a leakage current when the applied bias is higher than the reduction potential of the redox couple.

Figure 3B shows simulations and experiment of another ZnO QD film, but with ferrocenium present in solution, which has a reduction potential that is inside the band gap of ZnO. In this case, a reduction peak is observed as soon as the applied potential is high enough to inject electrons into the conduction band (around −0.85 V in Figure 3B). Once electrons can be injected into the ZnO, they can reach any ferrocenium species that are present in the film and reduce them, resulting in a sharp peak at the CB edge, as we reported before.^24^ However, no oxidation peak is observed, since the reduction potential is inside the bandgap the material. In terms of the Gerischer model (Figure 2), all the filled reductant energy states are below the CB edge of the ZnO, so no electrons would be transferred back to the ZnO even if the conduction band is completely empty. This means that the reduction of ferrocenium to ferrocene is also irreversible in this case. At potentials above the CB edge, an additional irreversible leak current is observed for the same reasons as mentioned for the cobaltocene system in Figure 3A. The reversibility of the redox reaction thus depends on the reduction potential compared to the conduction band edge of the QDs.

While this shows that irreversible CVs can be due to kinetically facile reactions with redox impurities with an *E*^*0*^ in the bandgap, it is unlikely that this explains the loss of electrons shown in Figure 1 for 2 reasons. First, the sharp peak at the CB edge that is due to the oxidation of such ideal impurities is not observed in experiments. Second, as shown below addition of oxygen makes CVs on ZnO films completely irreversible in experiment, even though its reduction potential is in the conduction band. To explain these observations, we next consider what happens if an electron transfer reaction to a redox impurity is coupled to an irreversible chemical reaction in an reversible electrochemical-irreversible chemical (E_r_C_i_) mechanism.

### Loss of Electrons to Oxidant, Followed by Irreversible Reaction

**Figure 4:**
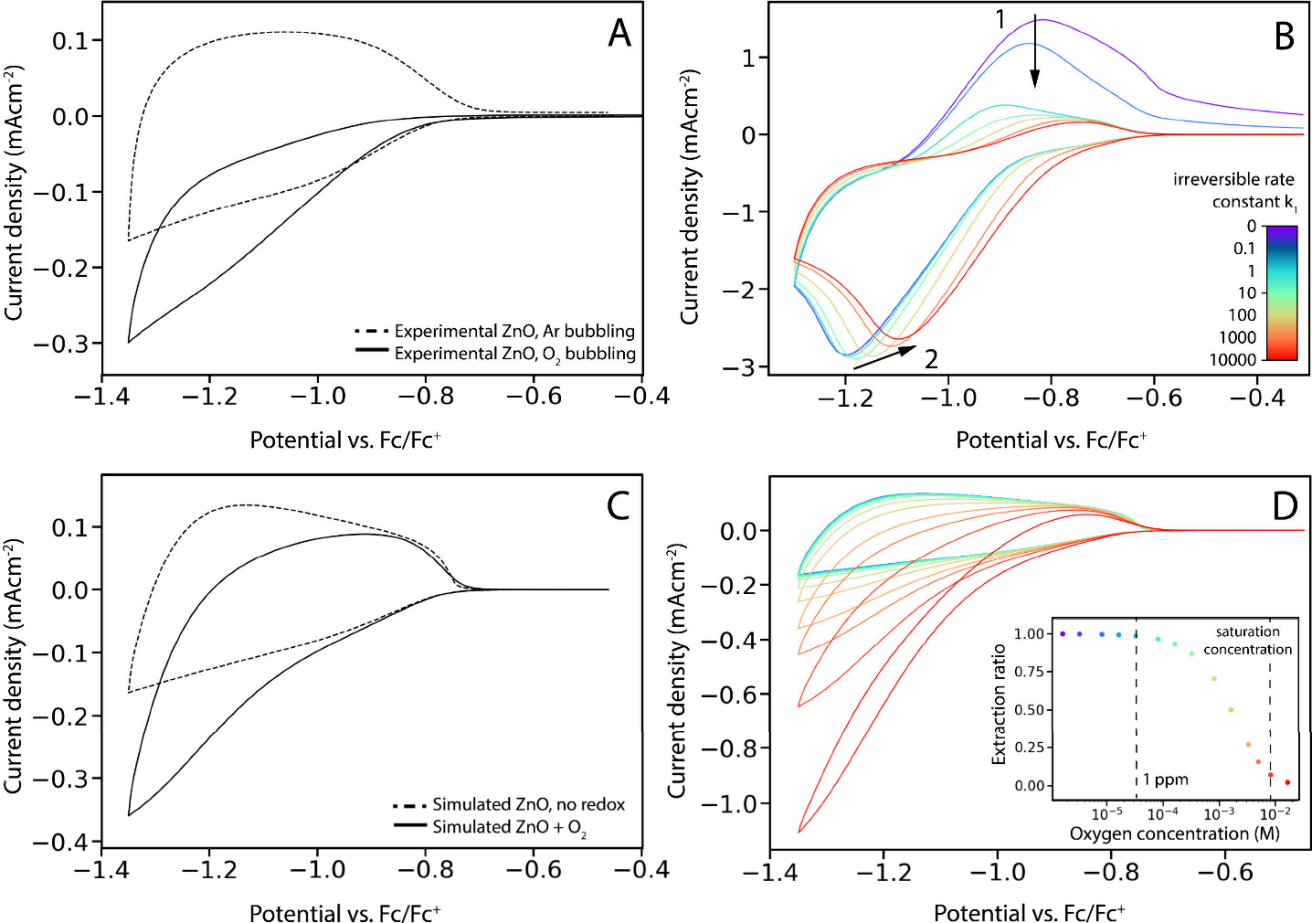
(A) Experimental CVs of a ZnO QD film with and without oxygen in the electrolyte solution (0.1 LiClO_4_ in acetonitrile) at a scan rate of 100 mV/s. QD diameter is 2.5 nm. (B) Simulated CVs of a ZnO QD film in the presence of an oxidant molecule following the coupled reversible-irreversible mechanism. As the rate of the irreversible reaction increases, reaction with the oxidant becomes more irreversible. This results in reduction of the oxidation peak (1) as well as a shift of the reduction peak to lower energies (2). (C) Simulated ZnO CVs with and without the addition of oxygen. Reduction of oxygen can only be successfully modeled by adjusting the Gerischer model to include a distribution of *E*^0^ values around the experimentally observed value of *E*^0^ = −1.2 V vs. Fc/Fc^+^. Simulated oxygen concentration = 3.3 mM, diffusion coefficient = 2.6 × 10^–6^ cm^2^s^–1^. (D) Simulated ZnO CVs with oxygen at different concentrations, ranging from 1.6 × 10^–6^ M (purple) to 1.6 × 10^–2^ M (red), diffusion coefficient = 2.6 × 10^–6^ cm^2^s^–1^.

The most relevant potential oxidant molecule in real systems is molecular oxygen itself, especially when permanent electrochemical doping is considered.^14^ Indeed, adding oxygen to the electrolyte solution makes CVs on ZnO QDs irreversible. In Figure 4A, two CVs on ZnO QD films are presented, after bubbling either argon or O_2_/N_2_ 0.21/0.79 gas through the electrolyte solution. The addition of molecular oxygen reduces the extraction ratio from 0.92 to 0.0. This shows that molecular oxygen acts as a oxidant and makes charge injection into QDs irreversible; it does not necessarily mean that the reason CVs on QDs are typically irreversible is due to oxygen.

In aprotic media, reduction of oxygen occurs through single electron reduction to the superoxide radical:
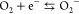


Under strict anhydrous conditions this reaction has been shown to be ideally reversible in acetonitrile, with a reduction potential very similar to cobaltocene/cobaltocenium at ∼ −1.2 V vs. Fc/Fc^+^.^25^ Based on this it would be expected that the ZnO CV with bubbled oxygen would resemble those with cobaltocenium in solution, but we instead observe irreversible behavior. To investigate this difference, we performed CVs on bare ITO and bare glassy carbon (GC) electrodes after bubbling O_2_/N_2_ 0.21/0.79 through the electrolyte in order to saturate the oxygen concentration. These CVs are shown in Figure S1 in addition to the same scans but with argon bubbled to remove any oxygen from solution. Rather than reversible reduction and oxidation of oxygen, we observe only a fully irreversible reduction peak when oxygen is present in the solution, with a width of >0.3 V. This is likely explained by reactions of the oxygen radical that is formed upon reduction with impurities in the solvent, for example trace amounts of water that are present in as-purchased solvents (∼10 ppm even for anhydrous solvents), or contaminations on the electrodes.^26,27^ To observe fully reversible reduction of oxygen, both a very clean (water-free) solvent and electrode are required, since O_2_^–^ is known to react further with H_2_O and H^+^ impurities.^27^ When considering reactions with QD films, many different contaminations can be present which can react with superoxide radicals, especially ligands (hydroxides on ZnO, carboxylates/amines on CdSe, PbS and InP). Ideal reversible reduction of oxygen is therefore unlikely, so we model the oxygen reduction as a reversible electrochemical-irreversible chemical (E_r_C_i_) reaction, as shown in the reaction scheme below.
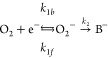


The first step is a reversible single-electron reduction with forward and backward rate (*k*_*1f*_*, k*_*1b*_) dependent on the applied potential, modeled using Gerischer kinetics. The second step is irreversible chemical conversion of the reduced species. For the second step we used a constant rate *k*_*2*_ as an input parameter for the simulation.

Figure 4B shows an example of a simulation incorporating E_r_C_i_ reaction kinetics for different values of *k*_*2*_. When the irreversible rate constant is low, the result is comparable to the simulation shown in Figure 3A, with a reversible redox couple in the conduction band. As *k*_*2*_ increases, the reaction becomes more irreversible. The oxidation peak disappears (marked by 1 in Figure 4B), as all the reduced species are consumed before they can be oxidized again. The reduction peak also shifts to lower potentials with increasing *k*_*2*_ (marked by 2). This occurs when the irreversible part of the reaction becomes so fast that any reduced species that are formed are immediately reacted away, disturbing the equilibrium of the reversible step. These results resemble those on E_r_C_i_ reaction kinetics on flat electrodes.^28^ The simulations with the E_r_C_i_ reaction mechanism at a high *k*_*2*_ closely resemble the results from the experimental oxygen reduction on bare electrodes (Figure S1).

However, even employing the E_r_C_i_ reaction mechanism, the simulations do not yet fully resemble the experimental CVs of ZnO in oxygenated electrolyte. In the simulations in Figure 4B, a clear peak is observed in the cathodic current, which is not seen in the experiments in Figure 4A. A similar effect is observed when comparing the experimental CVs in oxygenated electrolyte on the glassy carbon and ITO electrodes, as the reduction peak on the ITO electrode appears at more negative potentials and is also much broader than on the glassy carbon. Using simulations with a wide range of parameters, we show that this widening of peaks cannot be explained with only the Gerischer model of charge transfer. An extended discussion of these simulations can be found in Figure S2 in the Supporting Information. In short, a broad reduction peak is observed in the experiments (both on bare ITO and on the ZnO QD film), which implies a wide distribution of oxidant states and thus a large reorganization energy λ. However, a large λ value in the Gerischer model would also push up the oxidant states to higher energy values, since the separation between the peaks of the oxidant/reductant states distribution is governed by λ as well (Figure 2). This would entail that the reduction would be observed only at much higher energy values and thus much more negative applied potential, around 2.5 V vs. Fc/Fc^+^, while this is clearly not the case in the experimental data. It thus follows that the experimental reduction of oxygen on ZnO and ITO cannot be modeled by 1-electron transfer Gerisher kinetics. One reason for this observed broad reduction peak could be that oxygen is reduced at different reaction sites on the ZnO QD or ITO surface, leading to a distribution of E^0^ and k^0^ values. Another reason could be an electrostatic potential variation on the ITO surface, due to the relatively high sheet resistance of the electrode material, which has been known to induce peak broadening in ITO electrodes. We simulated this peak broadening by considering a distribution of E^0^ values around −1.2 V vs. Fc/Fc^+^ instead of just one single E^0^ value, resulting in an increase of the width of the Gaussian distribution of energy states of the oxidant molecule. This results in the simulations shown in Figure 4C which fit the experimental data reasonably well. Choosing a range of k^0^ values would have the exact same effect on the distribution of energy states, leading to the same results.

Based on our experiments and simulations, the irreversible charging response of semiconductor NC films in the presence of oxygen can thus best be explained by the reversible reduction of molecular oxygen, followed by an irreversible chemical reaction of the formed superoxide radical.

### Reduction of the Semiconductor NC Material

The loss processes discussed are dependent on the presence of contaminations, which can feasibly be avoided. In fact, reducing the concentration of contaminants to below 1 ppm practically eliminates side reactions, as shown by simulations in Figure 4D. However, some electrochemical side reactions are intrinsic to the material, for example reduction of lattice ions, surface ions, or ligands. If these reactions are irreversible and occur with the ions in the crystal lattice they may lead to cathodic decomposition of the materials. If they are limited to the surface,^29^ they may change the photoluminescence by inducing trap states without fully decomposing the materials.^30,31^

A likely candidate for cathodic decomposition reactions is the reduction of the metal ion in semiconductor materials to neutral metal. For example, this reaction probably occurs in lead-perovskite and PbS nanocrystals, resulting in clear deposition of metallic lead.^12,23^ Similarly, DFT calculations have suggested that indium reduction can take place in InP QDs when they are charged.^30^ The addition of a shell around QDs helps protect them against cathodic decomposition if the ions in the shell have more negative standard reduction potentials (e.g., Zn^2+^ is more stable than Cd^2+^), and may allow (semi)stable electron injection into QD materials.^30,31^ Especially ZnS shells greatly improve the stability under negative potentials.^11^ Surface modifications also have a large influence on the reduction potential of the cations of QDs.^16^ Since the surface ions are the first to undergo reduction, increasing their reduction potential can improve the stability of the QDs to cathodic decomposition.

**Figure 5:**
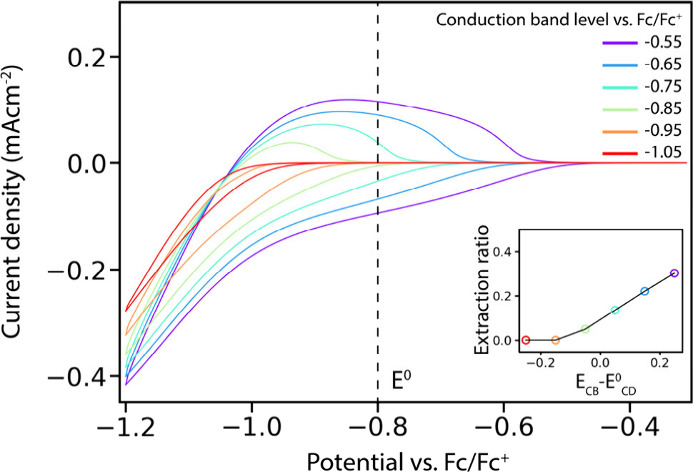
Simulated CVs of the charging of a QD film in the presence of a material reduction reaction with *E*^*0*^_*CD*_ = −0.8 V vs. Fc/Fc^+^. Different conduction band edges were used ranging from −0.5 to −1 V vs. Fc/Fc^+^. The inset shows the extraction ratios for different the conduction band edge levels compared to the *E*^*0*^_*CD*_. As the conduction band edge becomes more negative in energy, the charging becomes less reversible.

To model how such material reduction reactions influence CV measurements we again invoke Gerischer-kinetics and the E_r_C_i_ mechanism, but now considering species are not mobile (μ = 0) and with a high concentration (0.5 M). We model the effect of a reduction reaction on different materials by setting a constant E^0^ value for the reduction (−0.8 V vs. Fc/Fc+), while varying the energy of the CB. These simulations are shown in Figure 5.

As the conduction band edge becomes higher in energy than *E*^*0*^_*CD*_, the charging of the QDs becomes less reversible. When the CB edge is more than >0.1 eV higher in energy than the decomposition potential, fully irreversible charging is observed (extraction ratio = 0). In this case the simulated curves closely resemble experimental CVs on CdSe and InP QD films shown in Figure 1. Since the amount of oxidant contaminations in the electrolyte is typically low (which can be measured by running a CV on a blank electrode such as in Figure S1), material reduction is the most likely cause of irreversible behavior in experimental charging of semiconductor NC films. The simulations in Figure 5 confirm that material reduction can indeed lead to the observed irreversible charging in CVs on CdSe and InP QD films. Since the optical properties of these materials are often not permanently changed by electrochemical charging, the most likely candidate for reduction reactions are surface species (ligands or surface ions),^29^ while the inside of the semiconductor NCs remains unaffected.

Strategies to improve the stability of charges in the semiconductor NCs (and thereby the reversibility) therefore include surface modifications^16^ and/or the growth of shells. Another way to improve stability may be to shift the energy levels of the semiconductor NCs.^23^Figure 5 shows that even if *E*^*0*^_*CD*_ is inside the conduction band, when the electrochemical potential becomes too negative, material reduction can still occur. When the CB is filled up beyond the CB edge, the higher-energy electrons can still partake in electrochemical reduction reactions. The lowest available *E*^*0*^_*CD*_ will therefore put an upper limit on the concentration of charges that can stably be injected into a semiconductor NC material.

In many semiconductor NC materials, (electrochemical) hole injection has proven much more challenging than electron injection. This can mostly be ascribed to anodic decomposition reactions, as the *E*_*0*_ of anodic decomposition is above the valence band level in many materials,^32^ and thus anodic decomposition happens as soon as holes are injected into the semiconductor NCs. In devices such as QD-LEDs, hole injection is also generally more difficult than electron injection, even with the help of hole injection layers.^33,34^ With the simulations presented here, hole injection could be modeled in the same way as electron injection, providing more insight into this topic. In addition, similar analysis and arguments can be made for the processes resulting in loss of holes from semiconductor NCs. These types of simulations and analyses could help improve stability of semiconductor NC devices.

## Conclusion

In this work we model loss processes in electrochemically charged semiconductor nanocrystal films using drift-diffusion simulations and Gerischer kinetics. We consider both reactions with redox-active impurities and with the semiconductor NC materials themselves. The results are compared to experimental cyclic voltammetry on films of InP QDs, CdSe QDs, PbS QDs and ZnO QDs. The latter serve as an experimental model system, whereto we added controlled amounts of redox species.

We show that the presence of oxidant molecules in solution results in an additional reduction peak observed in CVs. If the reduction potential is above the conduction band of the semiconductor NC material, the reduced species can be oxidized again, and charging of the semiconductor NC remains reversible. When the reduction potential resides in the semiconductor NC band gap, however, reduction of the oxidant is irreversible and leads to loss of electrons from the semiconductor NCs. Loss of electrons to oxygen is best modeled using a reversible electrochemical-irreversible chemical reaction mechanism, because oxygen reduction is irreversible due to chemical reactions of the superoxide radical that is formed upon reduction. Reduction reactions of the semiconductor NC materials themselves are modeled by introducing a high concentration of immobile redox species in the semiconductor NC film. We show that material reduction reactions on the nanocrystal surface are the most likely candidate to explain the irreversible electrochemical behavior of InP and CdSe QD films, while for ZnO and PbS QD films, a small amount of dissolved oxygen (∼1 ppm) is responsible for the loss of electrons on longer time scales.

## Methods

All experimental procedures and measurements were performed in a nitrogen-filled glovebox (O_2_ < 0.5 ppm, H_2_O < 0.5 ppm) unless otherwise noted.

### Materials

Zinc acetate (99.99%), potassium hydroxide (KOH, 99.99%), anhydrous ethanol (max. 0.01% H_2_O), anhydrous methanol (≥99.8%), and anhydrous hexane (95%), Lead(II)oxide (PbO, 99.999%), 1-octadecene (ODE, 90%, degassed *in vacuo* at 120 °C for 1 h), bis(trimethylsilyl) sulfide (TMSS, synthesis grade), cadmium oxide (CdO, 99.99%), selenium powder (Se, 99.99%), trioctylphosphine (TOP, 97%, degassed *in vacuo* at 120 °C for 1 h), octadecylamine (ODA, 99%), trioctylphosphine oxide (TOPO, 90%), anhydrous methyl acetate (99%), anhydrous toluene (99.8%), indium(III) chloride (99.999%), zinc(II) chloride (>98%), tris(diethylamino)-phosphine (97%), lithium perchlorate (LiClO_4_, 99.99%, dry), 1,2-ethanedithiol (EDT, 98%), 1,7-diaminoheptane (7DA, 98%) ferrocenium hexafluorophosphate (97%, dried under vacuum), anhydrous acetonitrile (99.8%) and anhydrous methanol (99.8%) were purchased from Sigma-Aldrich. Oleic acid (OA, extra pure) was purchased from Thermo Fischer Scientific. Anhydrous oleylamine (80–90%) was purchased from Acros Organics. 1-carboxycobaltocenium hexafluorophosphate (dried under vacuum) was purchased from MCAT.

### ZnO QD Synthesis

The ZnO QDs were synthesized by a previously reported procedure.^24^ Zinc acetate (0.628 g) was dissolved in anhydrous ethanol (50 mL) by heating the solution to 60 °C while stirring. When dissolved, a solution of KOH (0.351 g) in anhydrous methanol (5 mL) was added dropwise (ca. 1 drop per second), and the solution was taken of the heat. The ZnO QDs were isolated from the reaction mixture by adding hexane until the solution became turbid. The mixture was centrifuged, the supernatant removed, and the QDs redispersed in 6 mL of ethanol. The QD dispersion was stored at −20 °C. The diameter of ZnO QDs used in experiments presented in Figures 3 and 4 was determined by transmission electron microscopy (TEM, Figure S3) to be 2.5 nm. The diameter of ZnO QDs used in experiments presented in Figure 1 was determined to be 3.8 nm by use of a sizing curve (ABS peak at 340 nm).^35^

### PbS QD Synthesis

PbS QDs were synthesized following a previously described procedure.^36^ Lead(II) oxide (90 mg) was dissolved in OA (0.25 mL) and ODE (3 mL) by heating *in vacuo* to 100 °C for 1 h. The temperature was then set the temperature to 150 °C, and a solution of TMSS (42 μL) in ODE (0.75 mL) was injected under a nitrogen atmosphere. The heating mantle was lowered away from direct contact with the reaction flask immediately after injection of the TMS solution and allowed to cool to room temperature. The PbS QDs were isolated from the reaction mixture by adding acetone until the solution became turbid, centrifuging the mixture, removing the supernatant and redispersing in 8 mL of hexane. The diameter of the PbS QDs was determined to be 5.5 nm by TEM imaging (Figure S3).

### CdSe Synthesis

CdSe QDs were synthesized according to a previously reported procedure.^11^ 0.077 M Cadmium oleate (Cd-oleate) solution was synthesized by dissolving 0.367 g CdO in 3.68 g OA and 25.9 g ODE. The mixture was first degassed *in vacuo* at 110 °C for 1 h and then heated to 250 °C under nitrogen atmosphere until a transparent solution was formed. Then it was cooled down to 110 °C and degassed again for 1 h. Afterward, the reaction was cooled to room temperature. 0.75 M selenium precursor (Se-TOP) was prepared by heating up a mixture of 1.42 g Se, 7.5 g TOP and 11.9 g ODE to 60 °C until the complexation was completed. In a 100 mL three-neck round-bottom flask, 3.2 g ODA and 1.11 g TOPO was heated to 140 °C and degassed for 1.5 h under vacuum. The mixture was placed under nitrogen atmosphere, and 5.2 g 0.75 M Se-TOP solution was added into the flask and the reaction was heated up to 300 °C. 4.9 g 0.077 M Cd-oleate solution was swiftly injected into the flask. The temperature was subsequently kept at 280 °C for 4 min. The reaction was quenched to 60 °C. To purify the CdSe QDs, anhydrous methyl acetate and anhydrous methanol with a ratio of 5:1 was added to the reaction mixture, followed by centrifugation at 3354 g. The supernatant was discarded and the residue redispersed in anhydrous toluene. This purification procedure was repeated once. The diameter of the CdSe QDs was determined to be 4 nm by TEM imaging (Figure S3).

### InP Synthesis

InP QDs were synthesized as reported previously.^37^ 100 mg (0.45 mmol) of indium(III) chloride and 300 mg (2.20 mmol) of zinc(II) chloride were mixed in 3 mL (9.10 mmol) of anhydrous oleylamine in a 25 mL flask. The mixture was stirred and degassed at 120 °C for an hour and then heated to 180 °C under inert atmosphere. Upon reaching 180 °C, 0.50 mL (1.83 mmol) of tris(diethylamino)phosphine, transaminated with 2 mL (6.07 mmol) of anhydrous oleylamine, was quickly injected in the reaction mixture described above and the InP nanocrystal synthesis proceeded for 30 min. The synthesized InP QDs were purified using anhydrous ethanol. The edge length of the QDs was determined to be 2.7 nm using a sizing curve (ABS maximum = 530 nm).^38^

### TEM Imaging

Transmission electron microscopy images were acquired using a JEOL JEM1400 transmission electron microscope operating at 120 keV.

### Experimental Cyclic Voltammograms

Experimental cyclic voltammogram measurements were performed using an Autolab PGSTAT128N potentiostat. A three-electrode setup was used, with a platinum sheet as the counter electrode, a silver wire as the pseudo reference electrode and an indium tin oxide (ITO)-coated glass plate as the working electrode. The pseudo reference electrode was referenced relative to 1 mM ferrocenium hexafluorophosphate in 0.1 M LiClO_4_ in acetonitrile (at −5.0 vs. vacuum level), which is used as the reference potential for all measurements. The electrolyte was 0.1 M LiClO_4_ in anhydrous acetonitrile for all experiments.

### Cyclic Voltammograms of Different Materials

QDs were deposited on the ITO working electrode using different techniques: ZnO QDs were dropcast on top of ITO-coated glass (film thickness = 700 nm) and annealed at 60 °C for 1 h before the measurement. PbS QDs were dropcast on top of ITO-coated glass (film thickness = 2400 nm) and dried at room temperature. The films were then immersed in a ligand exchange solution (0.1 M EDT in anhydrous acetonitrile) for 1 min to replace the isolating oleate ligands and rinsed with anhydrous acetonitrile.^23^ CdSe QDs were spin-coated on ITO-coated glass (film thickness = 40 nm). The QD films were then immersed in a ligand exchange solution (0.1 M 1,7-diaminoheptane in anhydrous methanol) for 1 min to replace the oleate ligands on the surface. Films were washed with anhydrous methanol to remove any excess 7DA.^11^ InP QDs were spin-coated on ITO-coated glass substrates. Films were then submerged anhydrous acetonitrile with 0.1 M EDT overnight to completely exchange the native ligands. Films were rinsed with anhydrous acetonitrile to remove any excess EDT. When quantum dots are covered in long apolar ligands, on-film ligand exchange is crucial to allow electrochemical charge injection and movement of charge through the film. Replacing long apolar ligands by shorter ligands such as EDT or 7DA both removes the isolating ligand barrier and brings the QDs closer together, greatly increasing charge transport between quantum dots through charge hopping.^10^ A comparison of the film parameters can also be found in Table S1 in the Supporting Information.

### Cyclic Voltammograms on ZnO QDs with Ferrocene/Cobaltocene

ZnO QDs were dropcast on top of ITO-coated glass and annealed at 60 °C for 1 h before the measurement. Anhydrous acetonitrile with 0.1 M LiClO_4_ and 1 mM of either ferrocenium hexafluorophosphate or 1-carboxycobaltocenium hexafluorophosphate was used as the electrolyte solution.

### Cyclic Voltammograms on ZnO with Oxygen

ZnO QDs were dropcast on top of ITO-coated glass and annealed at 60 °C for 1 h before the measurement. A solution of 0.1 M LiClO_4_ in anhydrous acetonitrile was prepared. Through this solution either pure Argon (99.9999%) or dry O_2_/N_2_ (21%/79%) was bubbled for 20 min. The ZnO films on ITO, bare ITO electrode or bare glassy carbon electrodes were then submerged in the electrolyte solution and measurements were performed. Measurements involving oxygen-bubbled solutions were performed outside the glovebox.

### Drift-Diffusion Simulations

The drift-diffusion simulations used are an extension of earlier work.^17^ Full computational details can be found in the Supporting Information. In short, a 3-electrode electrochemical cell is modeled as a 1-dimensional system, divided numerically in 250–490 lamella. The cell consists of a working electrode (WE) with a semiconductor NC film (porosity = 50%^24^), an electrolyte which contains mobile ions and can also contain electrochemically reactive species, a counter electrode (CE) which is treated as a capacitor with infinite capacitance and a reference electrode (RE) in the middle of the cell (schematic in Figure 1A). To model the electron injection into the semiconductor NCs, we assume that the NCs in the first lamella are in Boltzmann equilibrium with the WE (Table S2). This assumption implies this step is never rate-limiting. The Poisson equation is solved for each time step, then the movement of all mobile species is calculated using drift-diffusion equations (Table S2). Reactions between electrons that are in the conduction band of the semiconductor NCs and electrochemically reactive species are governed by the Gerischer kinetic model^18^ (Supporting Information). The Gerischer model is a better fit than the Marcus model for this scenario, since it considers reactions involving all energy states in the semiconductor NCs, instead of considering only a single energy level. All parameters used in the various simulations presented here are listed in Table S3. Experimentally known parameters like the temperature, scan speed and electrolyte and reductant/oxidant concentration were set in the simulations mirroring the experiments that they correspond with. Other parameters (most notably the effective mobilities of species inside the QD film) were optimized while being restrained to their expected range by running multiple simulations (such as in Figure 4D) to fit the experimental data. When simulating the same film with and without the presence of oxidant/reductant, these parameters were first optimized to fit the experiment performed without oxidant/reductant, then kept the same for the simulation with the oxidant/reductant. Simulations were performed on the DelftBlue cluster.^39^ The C++ source code underlying the simulations as well as accompanying instructions are available on Github: https://github.com/RFUbbink.

## Supporting Information Available

Computational details, simulation parameters, and experimental and simulated cyclic voltammograms (PDF)
