# Supplementary material for: Where Do the Electrons Go? Studying Loss Processes in the Electrochemical Charging of Semiconductor Nanomaterials
Source: Chem Mater. 2025 Jan 13;37(2):736–45. doi: 10.1021/acs.chemmater.4c02998 (PMC11780746; doi:10.1021/acs.chemmater.4c02998)
Supplement: Supplementary file 1 — cm4c02998_si_001.pdf [file cm4c02998_si_001.pdf]

Supporting information for

Where do the Electrons Go? Studying Loss Processes in the Electrochemical Charging of Semiconductor Nanomaterials

*Reinout F. Ubbink†, Yan B. Vogel†, Maarten Stam†, Hua Chen†, Arjan J. Houtepen†**

† Optoelectronic Materials Section, Faculty of Applied Sciences, Delft University of Technology, Van der Maasweg 9, 2629 HZ Delft, The Netherlands

Additional computational details

A 3-electrode electrochemical cell is modelled as a 1-dimensional system. The 1-dimensional simulated space starts at the working electrode (WE), encompasses the semiconductor NC film and the electrolyte solution, and ends at the counter electrode (CE). The reference electrode (RE) is positioned halfway between the film/solution interface and the CE. To achieve high spatial resolution in the film/solution interface region without sacrificing computational performance, the space was divided in lamella non-uniformly (see Table S2 for resolution parameters). The simulator considers an initial state, which contains starting values of the concentrations of

electrons, anions, cations, and oxidant and reductant species for each lamella. It then determines the movement of these species over small time steps (*dt*, in the order of 1e-9 to 1e-7 s) based on the drift-diffusion equations (Table S1). The hole concentration is assumed to be zero as only negative applied potentials relative to the open circuit potential are considered in this work. During each time step, a midpoint method is used to solve the Poisson equation (Table S1) and to determine the spatial profile of the electrostatic potential for the next step. Boundary conditions of a regular 3-electrode system are enforced, so $\phi_{WE} - \phi_{RE} = V_{applied}$ and $\phi_{RE} = 0$ (with ϕ the electrostatic potential at a certain position), while the electrostatic potential at the counter electrode is allowed to float. The true potential at the RE (and thus the initial Fermi level of the system) at open circuit potential is arbitrary for the simulation, but for comparison with experiments was set to -4.7 V *vs.* vacuum = 0.26 V *vs.* SHE = -0.3 V *versus* ferrocene/ferrocenium (Fc/Fc^+) in acetonitrile. This value corresponds to the work function of ITO (used as working electrode) and is in accordance with the open circuit potential of experimental ZnO QD films.[1, 2] The initial state of the simulation always consisted of an uncharged semiconductor NC film, where the concentration of electrons was zero, and an electrolyte solution with a certain concentration of cations and anions, c^0. An infinite supply of ions was simulated by setting the concentration of both ions at the RE constant at c^0 during the simulation. This most closely mirrors the much larger 3-dimensional volume of electrolyte solution that is available in experiments. The semiconductor NC film was considered to have a porosity of 50%, with the pores filled with electrolyte solution at concentration c^0. An initial concentration of uncharged oxidant species can also be set. The initial concentration of oxidant species is considered to be constant throughout the simulated space, including the

semiconductor NC film. Oxidant species can react with electrons in the semiconductor NC film, becoming negatively charged reductant species in the process. Similar to the ions, an infinite supply of oxidant species is artificially created by setting its concentration at the reference electrode to a constant value. Reactions between the electrons in the semiconductor NC s and the oxidant and reductant species were modelled using Gerischer kinetics, explained in detail below.

The electron concentration in the first lamella of the film, in contact with the ITO electrode, is governed by a Fermi-Dirac equilibrium with the electrode (Table S1). As the applied potential becomes more negative, the value of the Fermi level, Fermi-Dirac integral and the concentration of electrons all increase in the first lamella. Any DOS function can be used as input in the simulator. The simulation parameters, including the DOS function, were set at the start of the simulation, after which only the applied potential was altered to obtain the CV curves shown in this work. Parameters used were optimized to most closely fit the corresponding experimental electrochemical data (see Table TS2). For performance reasons, the simulator was written in C++ and compiled using Microsoft Visual Studio. The simulator source code and accompanying instructions are available at github: github.com/RFUbbink/QDfilmsim.

Table S1. Comparison of properties of QD films used in experimental CVs of this work. QD diameter determination method is indicated for each material. Either average diameters were measured from TEM images, or a sizing curve was used to calculate the diameter from an absorbance measurement, in which case the sizing method is referenced.

Material (Figure)	ZnO (Figure 1)	PbS (Figure 1)	CdSe (Figure 1)	InP (Figure 1)	ZnO (Figures 3-5)
QD diameter	3.8 nm (ABS = 340 nm)[3]	5.5 nm (TEM)	4 nm (TEM)	2.7 nm (ABS = 530 nm)[4]	2.5 nm (TEM)
Ligands after ligand exchange	OH^-	1,2-ethandithiol	1,7-heptanediamine	1,2-ethandithiol	OH^-
Film thickness	700 nm	2400 nm	40 nm	Unknown	Unknown

Table S2. List of formulas employed in the simulator.

Formula	Name and purpose	Explanation of parameters
$J_n = nq\mu_n \frac{dV}{dx} - kT\mu_n \frac{dn}{dx}$<br>$J_c = cq\mu_c \frac{dV}{dx} + kT\mu_c \frac{dc}{dx}$<br>$J_a = aq\mu_a \frac{dV}{dx} - kT\mu_a \frac{da}{dx}$<br>$J_O = kT\mu_O \frac{dO}{dx}$<br>$J_R = \mathrm{R}q\mu_R \frac{dV}{dx} - kT\mu_R \frac{dR}{dx}$	**Drift-diffusion equations**<br>Calculate the currents of electrons (n), cations (c), anions (a), oxidant (O) and reductant molecules (R).	J: current density<br>q: elementary charge<br>V*: electrostatic potential level vs vacuum<br>μ: carrier mobility
$\frac{d^2V}{dx^2} = \frac{q}{\varepsilon_0\varepsilon_r}(n + a - c + R)$	**1D Poisson equation**<br>Calculate the potential profile over the space of the simulation.	ε_0, ε_r: vacuum and relative electric permittivity.
$n = \int_{E=Ec}^{\infty} g_c(E) \frac{1}{1 + e^{\frac{E-E_F}{kT}}} dE$	**Fermi-Dirac distribution**<br>Calculate the equilibrium concentration of electrons at the interface between the WE and the semiconductor NC film.	E: energy<br>E_c: conduction band level<br>E_F: current Fermi level (= intrinsic Fermi level - electrostatic potential)<br>g_c (E): density of states function of the material

* For electrons, the total potential relative to vacuum is instead used, i.e. *energy level in the DOS* vs. *vacuum* + *electrostatic potential*. In this way, the extra energy needed for electrons to occupy higher levels in the DOS at higher energies is taken into account in calculating the drift current. The energy level of electrons in the DOS is calculated from the electron concentration.

Gerischer kinetic model employed in the simulator

We are considering the reaction of an electron residing in the conduction band (CB) of a semiconductor material with some oxidant in solution. The effective transfer of the electron is from an energy state in de CB to the HOMO of the oxidant molecule. If the CB consisted of only one energy level, a Marcus rate could be calculated, however there are many different energy levels in the CB at varying levels of occupancy. For this reason, we calculate the reaction rate using the Gerischer kinetic model.[5] The oxidant molecule is modelled using a redox potential E^0 and a reorganization energy *λ*. The reorganization energy of a molecule is determined by the reorganization of solvent molecules around the oxidant once it accepts an electron and becomes

charged. Reorganization energy is typically large (order of 2 eV) for molecules where the charge is localized, as the solvent will reorganize a lot in reaction to the charge. For instance the reduction of O_2 to the O_2^- radical is associated with a large reorganization energy. Delocalized or shielded charges, such as in the ferrocene/ferrocenium couple, are associated with much smaller reorganization energies (order of 0.5 eV). The reorganization energy of solvent molecule orientation can be calculated with the following formula:

$$\lambda = \left(\frac{1}{2r_O} + \frac{1}{2r_W} - \frac{1}{r_O + r_W}\right) * \left(\frac{1}{\varepsilon_{opt}} + \frac{1}{\varepsilon_s}\right) \qquad (1)$$

With r_O and r_W the radii of reductant and oxidant, and ε_{opt} and ε_s the optical and static dielectric constants of the solvent. In our case r_O is the radius of the semiconductor NC, while r_W is the radius of the redox-active species.

Using these two parameters, an effective "density of states" can be calculated for the oxidant/reductant pair. Both the empty states W_O (corresponding to the oxidant) and filled states W_R (reductant) are modelled as a Gaussian distribution according to the formulas:

$$W_O(E,\lambda) = \frac{1}{\sqrt{4\pi kT\lambda}} e^{-\frac{(E-E^0-\lambda)^2}{4kT\lambda}} \qquad (2)$$

$$W_R(E,\lambda) = \frac{1}{\sqrt{4\pi kT\lambda}} e^{-\frac{(E-E^0+\lambda)^2}{4kT\lambda}} \qquad (3)$$

Figure 2 in the main text shows an example energy distribution next to an example DOS function of a semiconductor. Transfer of electrons can take place between states in the semiconductor and the redox couple that have the same energy (isoelectronic energy transfer). Electrons can thus transfer both from filled states in the CB to the oxidant molecule as well as from filled states in the reductant back to empty CB states. The conduction band occupancy is determined by the Fermi-Dirac distribution. The rate of electron transfer from semiconductor to oxidant k_f is then given by the integral over the product of filled CB states and (empty) oxidant states:

$$k_f = \frac{4\pi}{3}\rho\tau_O\nu\int_{-\infty}^{\infty} W_{oxidator}(E,\lambda)DOS(E)\,f(E)dE \tag{4}$$

And the rate of reverse process k_b, electron transfer from reductant to semiconductor, is given by the integral over the product of empty CB states and filled reductant states:

$$k_b = \frac{4\pi}{3}\rho\tau_O\nu\int_{-\infty}^{\infty} W_{reductor}(E,\lambda)DOS(E)\,(1-f(E))dE \tag{5}$$

With $f(E)$ being the Fermi-Dirac function. For a given number of electrons in the CB, as can be obtained from the simulation at each point in space, the Fermi level can be calculated, which in combination with the DOS function, E^0 and λ is enough to calculate the rate of electron transfer.

In practice, given the DOS function, E^0 and λ (which are all input parameters to the simulation), the reaction rate was calculated for a range of possible electron concentrations and saved in a lookup table before the simulation was started. This allowed quick lookup for the reaction rate

through interpolation, avoiding the calculation of the complex integral (which would need to be performed separately for each lamella in space as electron concentration can vary throughout the semiconductor). This is critical for simulation performance.

The prefactor of the integral contains 3 components. First a unit conversion factor that accounts for the fact that not all molecules can partake in the reaction, only those close to the semiconductor surface. We consider that an electron in a semiconductor NC can react with any molecules on the surface, where ρ is the semiconductor NC diameter. Second the overlap integral τ_O between the wave function of electrons in the semiconductor and oxidant molecule. Third the "attempt frequency" ν, i.e. the amount of times per second that an electron can make the transfer. The last two components vary between systems and are essentially unknowable until they are measured for a specific redox couple/semiconductor pair. The prefactor was thus left as a variable in the simulations, essentially serving as a base rate similar to k^0 in the Arrhenius equation. The effect of the prefactor on the reaction rate is explained in Figure S3. Since the E^0 of a redox couple can be determined experimentally and the λ can be calculated or estimated quite accurately, this leaves the prefactor as the only unknown variable in the simulation.

Table S3. Parameters used in the simulations. Known values like the temperature, scan speed and electrolyte and reductant/oxidant concentration were set in the simulations mirroring the experiments that they correspond with. Some parameters, like the exact electron mobility (as long as it is significantly higher than ion mobility in the QD film) or relative permittivity of the QD film do not have a significant effect on the simulation results, and were thus set to set values that were reasonable while not compromising the simulation performance. This leaves a few parameters that are unknown, but are expected to be within a certain range, most notably the QD film ion mobilities, DOS normalization factor and also the exact molecular oxygen concentration in Figure 4. These parameters were optimized while being restrained to the reasonable range mentioned above (by running multiple simulations, such as in Figure 4D) to fit the experimental data. If film thickness was known, it was set in the simulation to the known value. Otherwise it was also optimized in the same way. When simulating the same film with and without the presence of oxidant/reductant, these parameters were optimized to fit the experiment performed without oxidant/reductant, then kept the same for the simulation with the oxidant/reductant.

**Parameter**	**Fig. 3A**	**Fig. 3B**	**Fig. 4B**	**Fig. 4A, 4C, 4D**	**Fig. 5**
semiconductor NC film thickness	350 (nm)	700 (nm)	700 (nm)	300 (nm)	
Number of lamella (film)	25				
Lamella thickness (film)	13.92 (nm)	27.92 (nm)	27.94 (nm)	11.94 (nm)	
Number of lamella (film/solution interface)	65				
Lamella thickness (film/solution interface)	0.4 (nm)		0.3 (nm)	0.3 (nm)	

Distance between WE and CE	0.3 (mm)*	0.1 (mm)*	0.1 (mm)*	0.1 (mm)*	
Number of lamella (solution)	160			400	
Lamella thickness (solution)	~1875 (nm)	~625 (nm)	~625 (nm)	~250 (nm)	
Electron mobility	3e-10 ($m^2V^{-1}s^{-1}$)**				
Ion mobility (film)	3.5e-12 ($m^2V^{-1}s^{-1}$)			2e-13 ($m^2V^{-1}s^{-1}$)	
Ion mobility (solution)	5e-9 ($m^2V^{-1}s^{-1}$)		5e-10 ($m^2V^{-1}s^{-1}$)	5e-10 ($m^2V^{-1}s^{-1}$)	
Reductant/oxidant mobility (film)	2e-10 ($m^2V^{-1}s^{-1}$)	2e-9 ($m^2V^{-1}s^{-1}$)	1e-10 ($m^2V^{-1}s^{-1}$)	2e-10 ($m^2V^{-1}s^{-1}$)	0
Reductant/oxidant mobility (solution)	6e-8 ($m^2V^{-1}s^{-1}$)*	2e-8 ($m^2V^{-1}s^{-1}$)*	1e-10 ($m^2V^{-1}s^{-1}$)*	1e-8 ($m^2V^{-1}s^{-1}$)*	0
Reductant starting concentration	1.2 (mM)	1.9 (mM)	200 (mM)	3.3 (mM)	500 (mM)
Film porosity	50%[6]				
Temperature	300 (K)				
Relative permittivity electrolyte solution	37#				
Relative permittivity semiconductor NC film	10#				

* The distance between the counter and working electrodes in the experimental setup is on the mm scale (1-3 mm), but this is too large to accurately simulate. We instead reduced this distance to 0.1-0.3 mm. To compensate for this and still simulate the Ohmic drop correctly, the ion mobility in the solution was reduced by a factor of ~10 from the expected values.

** It has been shown that electrons have a much higher mobility in ZnO QD films than the one used here.[2] Increasing the electron mobility higher than ~2 orders of magnitude above the ion mobility in the film does not affect the results of the simulation however, as cation mass transport in the film quickly becomes the limiting factor. For performance reasons, the electron mobility was therefore kept at an unphysically low level.

Based on acetonitrile. The semiconductor NC film is assumed to be a mixture of a semiconductor NC material and the electrolyte solution, so an arbitrary in-between value was picked for its relative permittivity. The relative permittivity of either the film or solution does not affect the results of the simulation as long as the spatial resolution is sufficient and can be put to any desired value.

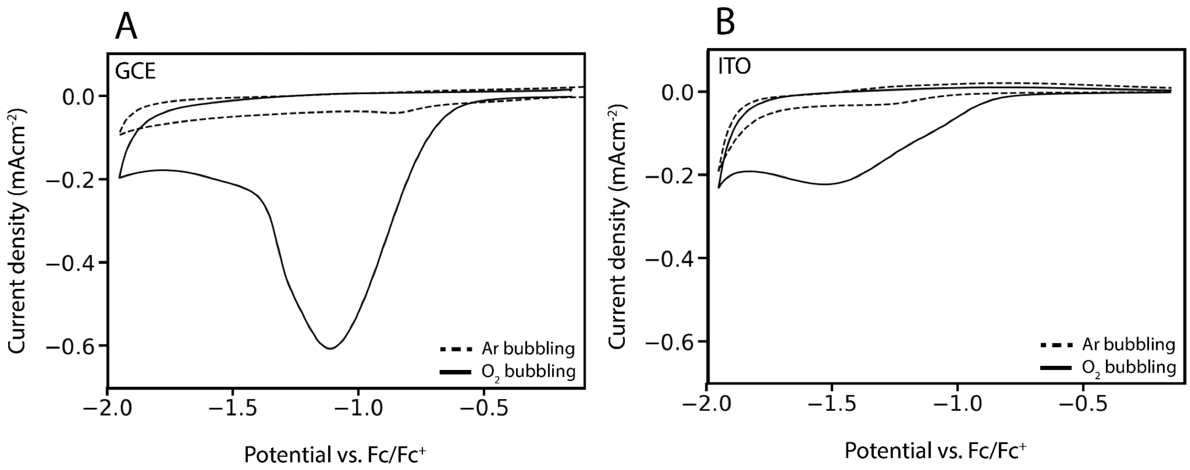

Figure S1. Experimental cyclic voltammograms performed on bare electrodes with either argon or O_2/N_2 0.21/0.79 bubbled through the electrolyte for 20 minutes. A) Glassy carbon electrode (GCE) and B) indium tin oxide (ITO) electrode. In both cases, an irreversible reduction peak is observed when oxygen is present. The reduction peaks observed for the ITO is broadened and shifted to more negative potentials compared to the glassy carbon.

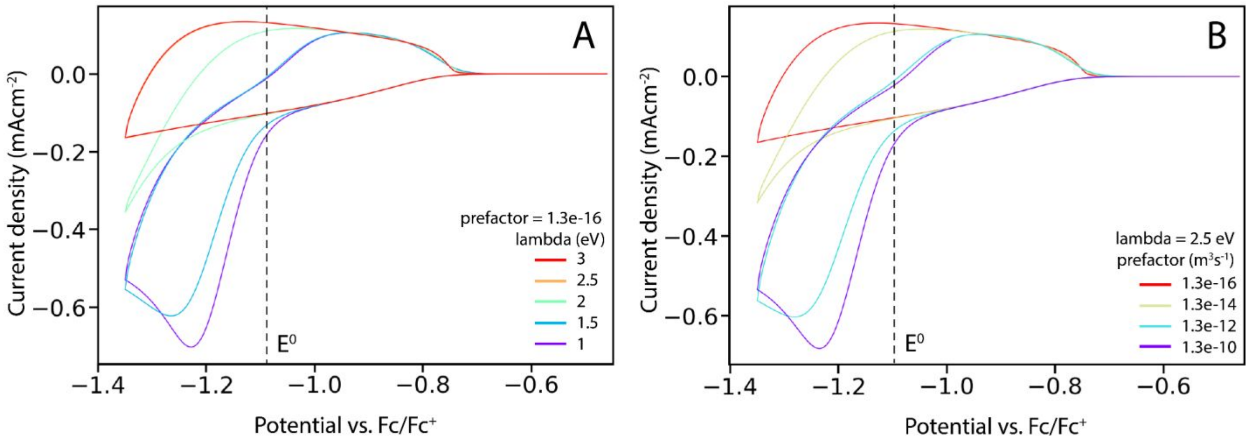

Figure S2. Simulated cyclic voltammograms of a semiconductor NC film in the presence of an oxidant following the coupled reversible-irreversible mechanism. The reduction potential is constant at -1.1 eV vs Fc/Fc^+, which is roughly where the oxygen to superoxide standard potential would be expected. It is impossible to simulate the experimental oxygen CVs (Figure 4B in the main text) with this reduction potential using only the unmodified Gerischer model. The expected parameters for the reorganization energy λ and prefactor would be 2.5 eV and 1.3e-16 m^3s^{-1} respectively. This results in no additional oxidative current, as the overpotential required to see significant current with these parameters is too large. Since the parameters are hard to determine/calculate based on experiments, both parameters were adjusted over a range of values to see if a fit could be obtained to the experiment. Increasing the prefactor or decreasing λ both results in lowering of the overpotential needed for reduction and thus an observed reduction current at low applied potentials. However, this reduction current increases exponentially, as is expected for the Gerischer model, and quickly reaches the point were diffusion limitation starts, leading to a peak in the current. This diffusion limitation is never observed in the experiments, which means

that the reduction current there does not increase exponentially. This means the experimental data cannot be fit with a Gerischer model.

References

1. Gudjonsdottir, S.; Van Der Stam, W.; Koopman, C.; Kwakkenbos, B.; Evers, W. H.; Houtepen, A. J., On the Stability of Permanent Electrochemical Doping of Quantum Dot, Fullerene, and Conductive Polymer Films in Frozen Electrolytes for Use in Semiconductor Devices. *ACS applied nano materials* **2019,** *2* (8), 4900-4909.
2. Gudjonsdottir, S.; Van Der Stam, W.; Kirkwood, N.; Evers, W. H.; Houtepen, A. J., The role of dopant ions on charge injection and transport in electrochemically doped quantum dot films. *Journal of the American Chemical Society* **2018,** *140* (21), 6582-6590.
3. Meulenkamp, E. A., Synthesis and growth of ZnO nanoparticles. *The journal of physical chemistry B* **1998,** *102* (29), 5566-5572.
4. Almeida, G.; van der Poll, L.; Evers, W. H.; Szoboszlai, E.; Vonk, S. J.; Rabouw, F. T.; Houtepen, A. J., Size-dependent optical properties of InP colloidal quantum dots. *Nano Letters* **2023,** *23* (18), 8697-8703.
5. Gerischer, H., Electron-transfer kinetics of redox reactions at the semiconductor/electrolyte contact. A new approach. *The Journal of Physical Chemistry* **1991,** *95* (3), 1356-1359.
6. Vogel, Y. B.; Stam, M.; Mulder, J. T.; Houtepen, A. J., Long-range charge transport via redox ligands in quantum dot assemblies. *ACS nano* **2022,** *16* (12), 21216-21224.
